# Developmental disruption to the cortical transcriptome and synaptosome in a model of *SETD1A* loss-of-function

**DOI:** 10.1093/hmg/ddac105

**Published:** 2022-05-09

**Authors:** Nicholas E Clifton, Matthew L Bosworth, Niels Haan, Elliott Rees, Peter A Holmans, Lawrence S Wilkinson, Anthony R Isles, Mark O Collins, Jeremy Hall

**Affiliations:** MRC Centre for Neuropsychiatric Genetics and Genomics, Division of Psychological Medicine and Clinical Neurosciences, Cardiff University, Maindy Road, Cardiff CF24 4HQ, UK; University of Exeter Medical School, University of Exeter, Exeter EX2 5DW, UK; Neuroscience and Mental Health Research Institute, Cardiff University, Maindy Road, Cardiff CF24 4HQ, UK; MRC Centre for Neuropsychiatric Genetics and Genomics, Division of Psychological Medicine and Clinical Neurosciences, Cardiff University, Maindy Road, Cardiff CF24 4HQ, UK; Neuroscience and Mental Health Research Institute, Cardiff University, Maindy Road, Cardiff CF24 4HQ, UK; MRC Centre for Neuropsychiatric Genetics and Genomics, Division of Psychological Medicine and Clinical Neurosciences, Cardiff University, Maindy Road, Cardiff CF24 4HQ, UK; MRC Centre for Neuropsychiatric Genetics and Genomics, Division of Psychological Medicine and Clinical Neurosciences, Cardiff University, Maindy Road, Cardiff CF24 4HQ, UK; Neuroscience and Mental Health Research Institute, Cardiff University, Maindy Road, Cardiff CF24 4HQ, UK; MRC Centre for Neuropsychiatric Genetics and Genomics, Division of Psychological Medicine and Clinical Neurosciences, Cardiff University, Maindy Road, Cardiff CF24 4HQ, UK; School of Biosciences, University of Sheffield, Western Bank, Sheffield S10 2TN, UK; MRC Centre for Neuropsychiatric Genetics and Genomics, Division of Psychological Medicine and Clinical Neurosciences, Cardiff University, Maindy Road, Cardiff CF24 4HQ, UK; Neuroscience and Mental Health Research Institute, Cardiff University, Maindy Road, Cardiff CF24 4HQ, UK

## Abstract

Large-scale genomic studies of schizophrenia implicate genes involved in the epigenetic regulation of transcription by histone methylation and genes encoding components of the synapse. However, the interactions between these pathways in conferring risk to psychiatric illness are unknown. Loss-of-function (LoF) mutations in the gene encoding histone methyltransferase, SETD1A, confer substantial risk to schizophrenia. Among several roles, SETD1A is thought to be involved in the development and function of neuronal circuits. Here, we employed a multi-omics approach to study the effects of heterozygous *Setd1a* LoF on gene expression and synaptic composition in mouse cortex across five developmental timepoints from embryonic day 14 to postnatal day 70. Using RNA sequencing, we observed that *Setd1a* LoF resulted in the consistent downregulation of genes enriched for mitochondrial pathways. This effect extended to the synaptosome, in which we found age-specific disruption to both mitochondrial and synaptic proteins. Using large-scale patient genomics data, we observed no enrichment for genetic association with schizophrenia within differentially expressed transcripts or proteins, suggesting they derive from a distinct mechanism of risk from that implicated by genomic studies. This study highlights biological pathways through which *SETD1A* LOF may confer risk to schizophrenia. Further work is required to determine whether the effects observed in this model reflect human pathology.

## Introduction

Schizophrenia is a leading cause of disability in young adults, and many patients remain insufficiently treated by current antipsychotics (1). Our understanding of the molecular mechanisms associated with risk for schizophrenia will be crucial for targeting new therapies. A string of recent genomic studies has unearthed hundreds of genomic loci each contributing small amounts of risk (2–5), improving power for the identification of relevant molecular pathways while complicating the recapitulation of their effects in model organisms. However, through advances in exome sequencing, a small number of single genes were identified containing a genome-wide excess of highly penetrant coding mutations in patients (6,7). This discovery greatly increases the feasibility of studying pathology relevant to schizophrenia in model organisms.

Rare loss-of-function (LoF) variants in the *SETD1A* gene, encoding SET Domain Containing 1A, confer substantial risk to schizophrenia and other neurodevelopmental disorders (6–8). The SETD1A protein catalyzes histone H3 (K4) methylation to mediate the expression of target genes. This lends support to the growing evidence that regulation of histone methylation is a point of convergence for genes conferring risk to neuropsychiatric disorders (9). SETD1A is required from very early in development for epigenetic control of the cell cycle and maintaining genome stability (10–13) but remains expressed in brain tissue throughout prenatal and postnatal life and appears to be required for normal neurite outgrowth, neuronal excitability and cognitive function (14–17). These observations suggest that *SETD1A* LoF may impact synaptic structure and function, and expose a mechanism through which risk to schizophrenia might be conferred.

Just as epigenetic control of gene expression is dynamic across development (18–21), the composition of the synapse varies considerably during brain maturation (22–24) as neurons migrate (25,26), form connections and mature. To explore the biological pathways through which *SETD1A* contributes to risk for schizophrenia, we quantified gene expression and synaptosome composition in the frontal cortex of mice carrying a *Setd1a* LoF allele at multiple prenatal and postnatal stages of development.

## Results

### Frontal cortex differential gene expression in *Setd1a*^+/−^ mice

**Figure 1:**
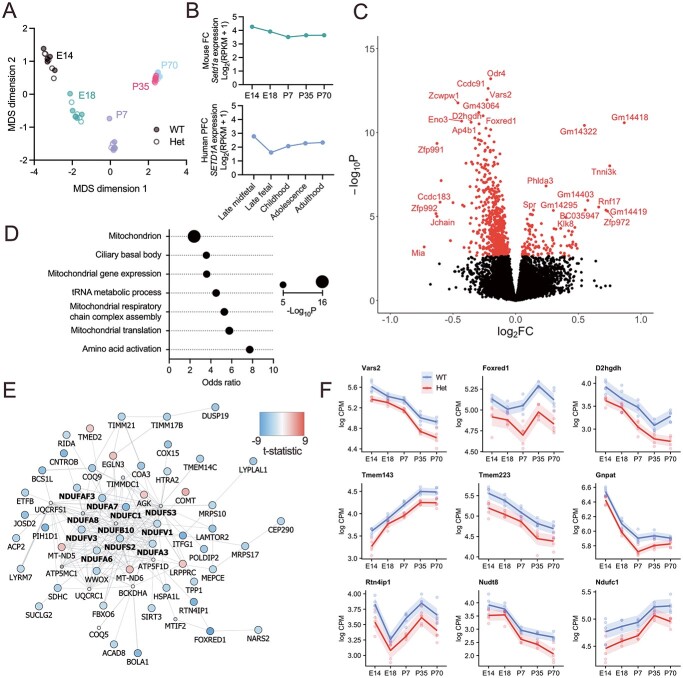
Transcriptomic effects of *Setd1a* LoF determined by RNA sequencing of mouse frontal cortical tissue across prenatal and postnatal development. (**A**) Multidimensional scaling (MDS) plot representing the similarity of sequencing libraries and clustering by timepoint. (**B**) Mean normalized frontal cortical expression of wild-type mouse *Setd1a* (top) or human *SETD1A* (bottom) across matched developmental timepoints. (**C**) Differential gene expression analysis contrasting *Setd1a*^+/−^ with wild-type samples, covarying for effects of age. Significantly differentially expressed genes (DEGs) are shown in red. (**D**) Enrichment of DEGs in genotype contrasts for functionally defined gene sets from the GO database. The size of the dot relates to the significance in Fisher’s exact test. (**E**) Protein–protein interactions among human orthologs of DEGs from genotype contrasts annotated by GO:0005739 *Mitochondrion*. Interaction data was obtained from GeneMANIA. Only proteins with interactions in the core network are displayed. Proteins forming part of the mitochondrial NADH:ubiquinone oxidoreductase respiratory complex I are shown in bold. Node color relates to the *t*-statistic in differential gene expression analysis. Smaller nodes indicate proteins inserted by GeneMANIA to improve the network but were non-significant in differential gene expression analysis. (**F**) Developmental expression of the top 9 DEGs in genotype contrasts, for wild-type and *Setd1a*^+/−^ samples. Shown is log counts per million (logCPM) ± standard error from embryonic day 14 (E14) to postnatal day 70 (P70). wild-type (WT); heterozygous (Het); frontal cortex (FC); prefrontal cortex (PFC); reads per kilobase of transcript per million mapped reads (RPKM); fold change (FC); counts per million (CPM).

Heterozygous knockout of *Setd1a* resulted in loss of approximately 50% Setd1a protein in cortical tissue compared with wild-type controls, reported previously (27). We performed RNA sequencing on 50 high-quality libraries (median RNA integrity = 9.35; Supplementary Material, Fig. S1, Supplementary Material, Table S1) from frontal cortex of *Setd1a*^+/+^ and *Setd1a*^+/−^ mice across five developmental timepoints (E14-P70; Fig. 1A). We analyzed the expression of 16 001 protein-coding genes expressed during at least one timepoint. In wild-type frontal cortex samples, *Setd1a* was expressed at all ages, consistent with human expression at matched developmental timepoints (Fig. 1B).

We used interaction analyses to identify any genes for which the effect of genotype differed by age. We observed no significant interaction terms after correction for FDR (Supplementary Material, Table S2) despite an overall negative correlation between the differential expression at E14 and E18, as indexed by the log fold change (Supplementary Material, Fig. S2). However, in genotype contrasts, controlling for age, we observed 734 genes differentially expressed (FDR < 0.05) between wild-type and *Setd1a*^+/−^ tissue (Fig. 1C; Supplementary Material, Table S3). The mutation led to considerably more downregulated genes (*N* = 616) than upregulated genes (*N* = 118).

Differentially expressed genes (DEGs) were enriched for seven Gene Ontology (GO) pathways predominantly relating to mitochondrial function (Fig. 1D, Supplementary Material, Table S4A). One hundred and forty-four differentially expressed genes intersecting with the GO:0005739 *Mitochondrion* term are listed in Supplementary Material, Table S4B. Mitochondrial pathways were only enriched among downregulated genes (Supplementary Material, Table S5). No GO pathways were significantly enriched in upregulated genes following Bonferroni correction (Supplementary Table S6). Using protein–protein interactions data, we identified a core network of proteins encoded by the DEGs, consisting of a central group from the mitochondrial NADH:ubiquinone oxidoreductase respiratory complex I and surrounding assembly factors (Fig. 1E). The rate of sequencing reads mapping to the mitochondrial genome did not differ by genotype (Supplementary Material, Fig. S3). In keeping with the lack of genotype-by-age interaction, the most significant mitochondrial DEGs were consistently downregulated across all the developmental timepoints examined (Fig. 1F).

We investigated the consistency of our results with previous studies of *Setd1a* haploinsufficiency. Six hundred and seventeen downregulated genes observed in a human neuroblastoma cell line following knockdown of *SETD1A* were also significantly enriched for GO:0005739 *Mitochondrion* genes (28). Of these, 454 genes had unique murine brain-expressed homologs, in which we observed an overlap of 68 genes with our downregulated gene set (Fisher’s exact Test: odds ratio = 4.76; *P* = 9.6 × 10^−22^). Conversely, 342 DEGs observed following *Setd1a* heterozygous knockout in 6-week-old mouse prefrontal cortex (14) showed proportionally less overlap (21 genes) with our DEGs, and did not exceed the chance level of overlap in Fisher’s exact Test (odds ratio = 1.44; *P* = 0.11). The same study also employed chromatin immunoprecipitation and sequencing (ChIP-seq) to identify direct targets of Setd1a on promoter or enhancer regions predicted to mediate gene expression (14). Using these data, we mapped Setd1a target peaks to promoter regions in 4970 genes and enhancer regions in 3738 genes. Notably, the GO term most significantly overrepresented among our DEGs following heterozygous *Setd1a* knockout—GO:0005739 *Mitochondrion*—was also strongly enriched among genes harboring promoter regions targeted by Setd1a (odds ratio = 1.42; *P*.bonferroni = 6.1 × 10^−4^). Furthermore, based on these data, 236 of our downregulated genes are targeted by Setd1a at promotor regions (Fisher’s exact Test: odds ratio = 1.30; *P* = 0.0015). This lends strength to the possibility that *Setd1a* LoF caused dysregulation of mitochondrial pathways through direct effects on gene regulation. Genes containing enhancer regions targeted by Setd1a were not enriched for *Mitochondrion* genes (odds ratio = 0.60; *P*.bonferroni = 1.0).

### Dysregulation of synaptosomal transcripts in *Setd1a*^+/−^ mice

**Figure 2:**
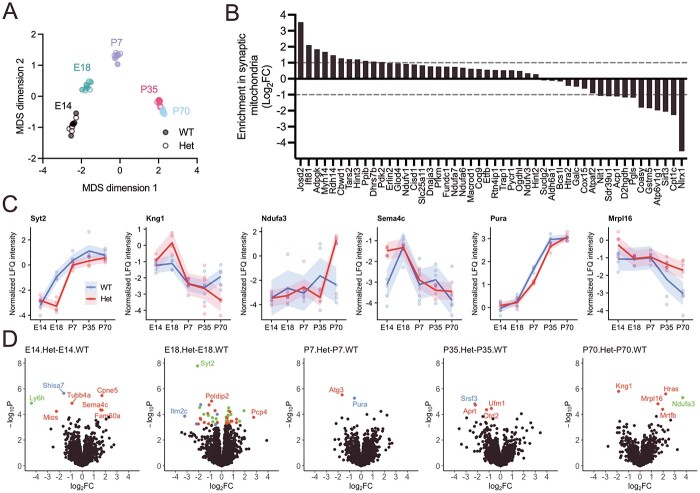
Alterations to the synaptosome caused by *Setd1a* LoF determined by mass spectrometry-based LFQ of isolated mouse synaptosomes across prenatal and postnatal development. (**A**) MDS plot indicating the clustering of synaptosome samples based on normalized LFQ intensity of each protein. (**B**) Relative abundance in synaptic versus non-synaptic mitochondria of proteins encoded by differentially expressed genes in genotype contrast. Mitochondrial proteomics data obtained from a previous study (29). (**C**) Wild-type and *Setd1a*^+/−^ synaptosomal protein abundance across development of differentially expressed proteins in primary genotype contrasts. Displayed is the normalized LFQ intensity ± standard error from embryonic day 14 (E14) to postnatal day 70 (P70). (**D**) Differential protein expression analyses contrasting *Setd1a*^+/−^ with wild-type synaptosomes at each timepoint independently. Colors indicate significantly differentially expressed proteins enriched in the PSD (blue), depleted in the PSD (green), or of similar abundance in the PSD compared with the total synaptosome/no data (red), based on published data (30).

To examine the effect of the *Setd1a* LoF allele on the regulation of synaptic components, we quantified changes in gene and protein expression relating to the synaptosomal fraction of frontal cortical tissue across the same timepoints (Fig. 2A). Using mass spectrometry-based label-free quantitation (LFQ) of isolated synaptosomes, we observed 3653 protein groups present in samples from at least one timepoint, after filtering. Within-sample comparisons of RNA and protein expression revealed good overall correlation (Supplementary Material, Fig. S4). Of the 734 DEGs from previous transcriptomic analysis of genotype effects, 127 (106 downregulated, 21 upregulated) encode proteins observed at the synaptosome (Supplementary Material, Table S7). More than half (58) of the downregulated synaptosomal genes were members of GO:0005739 *Mitochondrion*, indicating a strongly significant overrepresentation (odds ratio = 4.99; *P*.bonferroni = 2.9 × 10^−11^; Supplementary Material, Table S7). No other GO terms were significantly enriched among the downregulated synaptosomal genes. Again, no GO terms were enriched in the upregulated fraction (Supplementary Material, Table S8).

To predict whether *Setd1a* LoF preferentially impacted on mitochondria situated at the synapse, we compared our DEGs to published proteomic data (29) describing the relative abundance of proteins in synaptic versus non-synaptic mitochondrial proteomes. Forty-eight downregulated genes observed in genotype contrasts here encode proteins quantified from mitochondrial proteomes. Of these, 11 were enriched (log fold change > 1) in synaptic mitochondria and 11 were enriched in non-synaptic mitochondria (Fig. 2B). The ratio between these is no greater than the overall proportion of proteins enriched in synaptic mitochondria (odds ratio = 0.60; *P* = 0.92), suggesting the effects of *Setd1a* LoF on mitochondrial components were not specific to synapses but distributed between synaptic and non-synaptic compartments.

### Disruption to the synaptosomal proteome in *Setd1a*^+/−^ mice

We tested the effect of the *Setd1a*^+/−^ genotype on the synaptosomal proteome. To identify synaptosomal proteins for which the change in abundance over time was affected by *Setd1a* LoF, we contrasted the difference in protein expression between all pairs of consecutive timepoints in mutant and wild-type samples. The change in developmental expression of two proteins, Kng1 and Ndufa3, differed by genotype (Supplementary Material, Table S9). By examining each contrast, we observed that synaptosomal Kng1 intensity was significantly affected by genotype between E14 and E18 (*t* = 2.09, *P* = 0.042), E18 to P7 (*t* = −2.12, *P* = 0.040) and P35 to P70 (*t* = −4.92, *P* = 1.3 $\times$ 10^−5^). Ndufa3 was affected by genotype between P7 and P35 (*t* = −2.48, *P* = 0.017) and from P35 to P70 (*t* = 5.75, *P* = 8.3 $\times$ 10^−7^). Analyzing across all timepoints, six proteins were significantly altered by genotype (Supplementary Material, Table S10): Synaptotagmin-2 (Syt2), Kininogen-1 (Kng1), NADH dehydrogenase (ubiquinone) 1 alpha subcomplex subunit 3 (Ndufa3), Semaphorin-4C (Sema4c), Transcriptional activator protein Pur-alpha (Pura) and mitochondrial ribosomal protein L16 (Mrpl16) (Fig. 2C). Notably, transcripts encoding Ndufa3 were also differentially expressed in transcriptomic analysis of genotype effects described before (Supplementary Material, Table S2). The functions of these proteins are summarized in Table 1.

**Table 1:** Differentially expressed proteins in cortical synaptosomes of *Setd1a*^+/−^ mice compared with wild-type, controlling for age. Synaptic compartment localization was determined from a previous study (30)

Protein	Function	Synaptic compartment
Syt2	Mediates calcium-dependent synaptic vesicle exocytosis and neurotransmitter release	PSD-depleted
Kng1	Precursor to the proinflammatory peptides of the kallikrein-kinin system	No data
Ndufa3	Subunit of the mitochondrial respiratory chain complex I	PSD-depleted
Sema4c	Receptor for Plexin-B2, important for regulation of axon guidance, dendritic morphology and synapse formation	No data
Transcriptional activator protein Pura	DNA- and RNA-binding protein involved in transcriptional control and cytoplasmic RNA localization	PSD-enriched
Mrpl16	Nuclear-encoded subunit of mitochondrial ribosomes, required for protein synthesis within mitochondria	No data

To examine the effect of *Setd1a* LoF at different developmental stages more closely, we performed genotype contrasts at each age independently, yielding a set of differentially expressed synaptosomal proteins for each timepoint (Fig. 2D; Supplementary Material, Tables S11–S15). As a whole, differential protein expression, as indexed by the log fold change, was poorly correlated between different stages of development (Supplementary Material, Fig. S2), and with differential gene expression compared at each stage (Supplementary Material, Fig. S5). Significantly upregulated or downregulated proteins were further annotated by predictions of their relative abundance in the postsynaptic density (PSD) compared with the total synaptosome based on previously published data (30). At all timepoints, differentially expressed PSD-enriched proteins were downregulated in *Setd1a*^+/−^ samples compared with wild-type. Presynaptic protein, Syt2, was strongly decreased at E18, such that the developmental upregulation observed in wild-types was delayed in mutant cortex (Fig. 2C). To obtain more biological insight into the types of proteins affected, we performed pathway analysis of those differentially expressed at any timepoint (N = 63), using a background of all synaptosomal proteins. We observed no significantly enriched pathways after multiple testing corrections (Supplementary Material, Table S16). It is notable, however, that genes belonging to the top term by significance in DEG pathway analyses (GO:0005739 *Mitochondrion*) represented the highest proportion of differentially expressed proteins (19 proteins), with a nominally significant enrichment (odds ratio: 1.70; *P*.unadjusted = 0.043). Seventeen of these *Mitochondrion* proteins were differentially expressed at E18 (Supplementary Material, Table S12), 12 of which were downregulated.

### Genetic association with schizophrenia of transcripts and proteins disrupted by *Setd1a* LoF

We hypothesized that molecular pathways disrupted by *Setd1a* LoF also contribute risk for schizophrenia through enrichment for genetic association with the disorder. We tested this using case–control data from genome-wide association study (GWAS) (PGC3) and exome sequencing studies. Through gene set association analyses of DEGs and affected proteins across brain development, including subsets defined by membership of the synaptosome or GO:0005739 *Mitochondrion*, we observed no significant enrichment for genetic association with schizophrenia in any gene set, through common or *de novo* rare variants (Table 2).

**Table 2:** Genetic association with schizophrenia of transcripts and proteins disrupted by *Setd1a* LoF. Differentially expressed genes or proteins observed from the specified contrasts were tested for enrichment for genetic association with schizophrenia through common or rare variation. *P*-values follow conditioning on all expressed genes (RNA analyses) or all synaptosomal genes (protein analyses) and are uncorrected for multiple testing, unless specified. PSD enrichment or depletion was predicted based on published data (30). Differentially expressed gene (DEG); Bonferroni-adjusted *P*-value (*P.*adj)

Differential expression contrast	*N* genes/proteins	Schizophrenia common variant association	Schizophrenia *de novo* rare non-synonymous variant association
RNA: genotype effect (all timepoints)			
All DEGs	734	*β* = −0.071 *P* = 0.95	Rate ratio = 1.03 *P* = 0.39
Downregulated	616	*β* = −0.091 *P* = 0.97	Rate ratio = 0.96 *P* = 0.80
Upregulated	118	*β* = 0.052 *P* = 0.32	Rate ratio = 1.31 *P* = 0.11
Synaptosomal	127	*β* = −0.063 *P* = 0.73	Rate ratio = 0.52 *P* = 0.97
Non-synaptosomal	607	*β* = −0.028 *P* = 0.72	Rate ratio = 1.15 *P* = 0.12
DEGs belonging to GO:0005739 *Mitochondrion*	101	*β* = −0.15 *P* = 0.91	Rate ratio = 1.08 *P* = 0.75
Protein: genotype effects (any timepoint)			
All unique proteins	67	*β* = 0.049 *P* = 0.37	Rate ratio = 1.49 *P* = 0.11
Downregulated	35	*β* = 0.098 *P* = 0.30	Rate ratio = 0.88 *P* = 0.67
Upregulated	33	*β* = −0.025 *P* = 0.55	Rate ratio = 2.45 *P* = 0.019 (*P*.adj = 0.21)
PSD-enriched	8	*β* = −0.39 *P* = 0.88	Rate ratio = 2.71 *P* = 0.17
PSD-depleted	22	*β* = 0.23 *P* = 0.15	Rate ratio = 1.18 *P* = 0.44

Owing to the small number of significantly differentially expressed proteins, we performed an additional test of genetic association with schizophrenia in gene sets ranked by the probability of differential protein expression in the synaptosome, determined from the main effect of genotype. Gene sets ranked highest for differential protein expression were not enriched for association with schizophrenia through common variation or *de novo* rare variation (Supplementary Material, Fig. S6), suggesting that *Setd1a* LoF does not preferentially disrupt synaptosomal proteins that contribute additional genetic risk to schizophrenia.

## Discussion

Understanding the biological effects of highly penetrant genetic mutations conferring risk to schizophrenia is crucial for unraveling pathology and improving treatments. We modeled a heterozygous *Setd1a* LoF allele in mice and profiled RNA and protein from frontal cortical tissue across multiple pre- and postnatal developmental timepoints. The mutation caused downregulation of transcripts predominantly enriched for mitochondrial function, irrespective of age. Using mass spectrometry-based protein quantitation, we further examined the effects of the *Setd1a* variant on the constituents of the synaptosome and revealed subsets of proteins disrupted at each timepoint.

Transcriptomic data from *Setd1a*^+/−^ mouse cortex showed evidence of disruption to respiratory chain complex I, mitochondrial assembly proteins and mitochondrial translation. Disruption by *Setd1a* haploinsufficiency of mitochondrial and metabolic functions characterized by a downregulation of associated nuclear transcripts is consistent with previous studies in human neuroblastoma (28). Altered metabolism following *Setd1a* deletion was also observed in hematopoietic stem cells (10). Similarly, loss of other SET domain-containing proteins, including the Set1 ortholog, *Setd1b* and *Setd5*, induced downregulation of mitochondrial and metabolic pathways (31,32), together supporting a role of chromatin modifications by this protein family in regulating mitochondrial function. While further work is needed to establish the nature of this relationship, we report that promoter regions targeted by Setd1a (14) are enriched in genes with functional annotations related to mitochondria, thereby providing evidence of a direct causal relationship.

Oxidative phosphorylation in mitochondria supplies the high metabolic demand of synaptic activity in neurons, and it has been suggested that mitochondrial dysfunction could cause progressive developmental synaptic pathology in schizophrenia (33–37). Fast-spiking parvalbumin interneurons, which have been recurrently implicated in schizophrenia, contain high densities of mitochondria, are highly susceptible to oxidative stress, and may be particularly vulnerable to metabolic disruption (38–40). Mitochondrial dysfunction in schizophrenia is supported by a range of studies presenting transcriptomic, proteomic and metabolomic evidence of reduced mitochondrial activity, predominantly relating to components of respiratory complex I, in post-mortem brain, peripheral tissues and induced pluripotent stem cells from patients (41–49). Genetic studies show some evidence of a burden of patient copy number variants (CNVs) in mitochondrial genes (50), yet the largest schizophrenia GWAS to date reported no enrichment for common genetic association in mitochondrial pathways (5). Consistent with this, we observed no enrichment in differentially expressed mitochondrial genes for schizophrenia association conferred by common variants or *de novo* rare non-synonymous variants. Hence, through quantifying the biological effects of a highly penetrant schizophrenia risk variant, our study informs potential functional pathways of risk that are not illuminated by primary genetic studies alone.

From previous work, it has been suggested that Setd1a has additional roles in synaptic function and development, and its LoF leads to deficits in working memory (14,15). However, unlike these previous studies, we observed no enrichment of neuron-specific functional annotations among DEGs in mutant samples. After restricting our transcriptomic analysis to genes encoding proteins detected in the synaptosome, we found that downregulated transcripts remained enriched for mitochondrial function. Through proteomic analysis, we observed multiple downregulated mitochondrial proteins, principally at E18, coinciding with a critical period of neuronal maturation and synaptogenesis (51). This paralleled a delay in the developmental upregulation of presynaptic neurotransmitter release protein Syt2, suggesting abnormal synaptic maturation in *Setd1a*^+/−^ cortical samples. However, whether the effects on Syt2 and other (non-mitochondrial) synaptic proteins were primary or secondary to *Setd1a* or mitochondrial dysfunction is undetermined.

Despite the apparent disruption to mitochondrial pathways in the synaptosome, we found evidence that *Setd1a* haploinsufficiency impacted synaptic and non-synaptic mitochondria. Therefore, any metabolic consequences of the mutation may be equally likely to influence non-neuronal cell types and other cellular compartments not examined in this study. Poor overall correlation between differential protein expression in the synaptosome and tissue-wide differential gene expression also suggests that many of the transcriptomic effects of the *Setd1a* variant influence non-synaptic compartments. However, owing to the high metabolic demands of neurotransmission, synaptic systems may be more sensitive to small changes in mitochondrial activity than other cellular processes (52). Future work using tissue- or cell-specific omics may seek to further characterize cortical metabolic abnormalities caused by *Setd1a* LoF.

DEGs observed here in genotype contrasts exhibited poor overlap with those derived from a previous transcriptomic study of adult *Setd1a*^+/−^ mouse prefrontal cortex (14), which in turn were inconsistent with a third transcriptomic study of *Set1a* haploinsufficiency (15), together with the biological pathways annotated to them. While we extended the investigation to multiple developmental stages, we found no significant effect of age on the differential expression signature. Critically, each of these three studies were performed using different mouse models, and while each resulted in the reduction of Setd1a protein in frontal brain regions by approximately 50% and the induction of schizophrenia-related behavioral phenotypes (14,15,27), their effects on particular isoforms or compensatory mechanisms may have differed. Further differences in tissue extraction and library preparation methods could also contribute.

To conclude, our results give evidence of disruption to nuclear-encoded mitochondrial pathways in cortical tissue throughout brain development caused by modeling a *SETD1A* LoF allele that confers substantial risk to schizophrenia. Our findings therefore support the premise of mitochondrial perturbation in psychiatric pathology and expose biological consequences of genetic risk that are not themselves predicted by genetic association studies. We further highlight a subset of synaptic proteins that may be key to understanding neural dysfunction induced by this variant.

## Materials and Methods

### Subjects and tissue preparation

Mice carrying a heterozygous *Setd1a*^tm1d^ LoF allele, with mixed C57BL/6NTac and C57BL/6 J background, were generated using a knockout-first design and genotyped as described previously (27). Heterozygous males were paired with wild-type females to generate male experimental subjects at embryonic day 14.5 (E14.5), E18.5, postnatal day 7 (P7), P35 and P70 (*N* = 5 per genotype per timepoint). Timed matings, determined by plug checks, were used for embryonic timepoints. For P35 and P70 timepoints, offspring were weaned at P28 and housed in single-sex groups. All animals were provided with environmental enrichment, food and water *ad libitum* and maintained at 21°C and 50% humidity with a 12-h light–dark cycle. All procedures were conducted in accordance with the United Kingdom Animals (Scientific Procedures) Act 1986 (PPL 30/3375).

At embryonic timepoints, pregnant dams were killed and frontal brain regions immediately dissected from embryos. At postnatal timepoints, littermates were killed and frontal cortex dissected. Brain tissue was snap frozen before storage at −80°C until processing. Bilateral frontal cortices were homogenized using a Dounce homogenizer in Synaptic Protein Extraction Reagent (SynPER, Thermofisher). A fraction of the homogenized sample was taken forward for RNA extraction and the remaining used for synaptosome extraction.

### Synaptosome isolation

Synaptosomes were isolated from homogenized cortical tissue using the SynPER protocol, as per the manufacturer’s instructions. Briefly, following homogenization, samples were centrifuged at 1200 g for 10 min (4°C) and the pellet discarded. The supernatant was centrifuged again at 15 000 g for 20 min (4°C) to generate the synaptosome pellet. We resuspended the pellet in 2% SDS, 50 mm Tris pH 7.4 and heated at 70°C for 15 min to extract the protein. Samples were clarified by centrifugation at 20 000 g for 10 min.

### Transcriptomics

RNA was extracted using an AllPrep DNA/RNA micro kit (QIAGEN) before quantitation and checks for integrity, degradation and contamination. Samples with <0.5 μg total RNA were replaced. Library preparation and sequencing were performed by Novogene. cDNA libraries with 250–300 bp inserts were prepared using poly-A capture. A single batch of Illumina high-throughput sequencing was performed at 12Gb read depth per sample with 150 bp paired-end reads (~40 million paired-end reads).

Raw sequencing reads were trimmed of adapters using Trimmomatic (53) and passed through FastQC quality control (54). Reads were aligned to the mouse genome (GRCm38) with STAR (55) and mapped to genes using featureCounts (56). Processed read counts were filtered for protein-coding genes. EdgeR (57) was used to determine and exclude unexpressed genes, and perform trimmed mean of M values (TMM) normalization (58). Expressed genes were defined as having at least 10 counts per million in at least five samples. Differential expression analyses were performed with limma (59). In primary analyses, we tested for genotype effects that varied by age by fitting an age $\times$ genotype interaction, coding age as a five-level factor. In subsequent analysis, gene expression was regressed on genotype, covarying for age. False discovery rate (FDR) was corrected for using the Benjamini–Hochberg method.

Postmortem human prefrontal cortex *Setd1a* expression data across the lifespan was obtained from the BrainSeq Phase I database (http://eqtl.brainseq.org/phase1/) (60). Samples were filtered for individuals with no history of psychiatric condition. Raw gene counts were converted to reads per kilobase of transcript per million mapped reads (RPKM) and averaged across five developmental stages: late midfetal (17–23 post-conceptual weeks; *N* = 13), late fetal (24–37 post-conceptual weeks; *N* = 3), childhood (1–12 years; *N* = 16), adolescence (13–19 years; *N* = 47) and adulthood (20–85 years, *N* = 202).

### Quantitative mass spectrometry analysis

Fifty micrograms of were solubilized with 5% SDS, 100 mm tetraethylammonium bromide pH 8 and reduced using 10 mm Tris(2-carboxyethyl)phosphine with heating at 70°C for 15 min. Samples were alkylated with 20 mm Iodoacetamide for 30 min at 37°C. Protein was precipitated in solution, trapped and washed on S-trap micro spin columns (ProtiFi, LLC) according to the manufacturer’s instructions. Protein was digested using 5 μg trypsin sequence grade (Pierce) at 47°C for 1 h and 37°C for 1 h. Eluted peptides were dried in a vacuum concentrator and resuspended in 0.5% formic acid for LC-MS/MS analysis. Peptides were analyzed using nanoflow LC-MS/MS using an Orbitrap Elite (Thermo Fisher) hybrid mass spectrometer equipped with a nanospray source, coupled to an Ultimate RSLCnano LC System (Dionex). Peptides were desalted online using a nano trap column, 75 μm I.D.X 20 mm (Thermo Fisher) and then separated using a 120-min gradient from 5 to 35% buffer B (0.5% formic acid in 80% acetonitrile) on an EASY-Spray column, 50 cm × 50 μm ID, PepMap C18, 2 μm particles, 100 Å pore size (Thermo Fisher). The Orbitrap Elite was operated with a cycle of one MS (in the Orbitrap) acquired at a resolution of 120 000 at m/z 400, with the top 20 most abundant multiply charged (2+ and higher) ions in a given chromatographic window subjected to MS/MS fragmentation in the linear ion trap. A Fourier transform mass spectrometry (FTMS) target value of 1e6 and an ion trap MSn target value of 1e4 were used with the lock mass (445.120025) enabled. Maximum FTMS scan accumulation time of 500 ms and maximum ion trap MSn scan accumulation time of 100 ms were used. Dynamic exclusion was enabled with a repeat duration of 45 s with an exclusion list of 500 and an exclusion duration of 30 s. Raw mass spectrometry data were analyzed with MaxQuant version 1.6.10.43 (61). Data were searched against a mouse UniProt reference proteome (downloaded May 2020) using the following search parameters: digestion set to Trypsin/P, methionine oxidation and N-terminal protein acetylation as variable modifications, cysteine carbamidomethylation as a fixed modification, match between runs enabled with a match time window of 0.7 min and a 20-min alignment time window, LFQ was enabled with a minimum ratio count of two, minimum number of neighbors of three and an average number of neighbors of six. A protein FDR of 0.01 and a peptide FDR of 0.01 were used for identification level cut-offs based on a decoy database searching strategy. This protocol yielded synaptic proteomes with comparable composition to those observed previously in mice (30).

Protein groups were converted to single proteins by prioritizing those explaining the most data. These 5142 proteins were filtered to include only those detected in at least four of five samples from at least one experimental group, giving 3710 proteins for analysis. Raw LFQ intensity values were log converted and scaled by median intensity normalization. Missing values were imputed from a normal distribution (mean = μ−1.8; standard deviation = σ $\times$ 0.3). Genotype contrasts were performed using limma (59). In primary analyses, all within-age genotype contrasts were tested in the same linear model to determine the mean effect of genotype across development for each protein. In interaction analyses, the difference in protein intensity between all pairs of consecutive time points was contrasted between wild-type and *Setd1a*^+/−^ samples, as described previously (62). For any significant interactions following correction for FDR (*P* < 0.05), the interaction terms from each pair of consecutive time points were extracted individually to identify specific periods when the protein is affected by genotype. In secondary analyses, genotype contrasts were performed at each age independently.

Data describing the relative abundance of proteins in synaptic versus non-synaptic mitochondria were acquired from a study of neuronal bioenergetic control in adult rat forebrain (29).

The localization of proteins to presynaptic or postsynaptic fractions of the synaptosome was predicted *in silico* using a previous report of synaptic protein enrichment or depletion in PSD compared with synaptosome preparations from mouse brain (30).

### Pathway analysis

Functional annotations of genes were compiled from the GO database (June 8, 2021), excluding gene annotations with evidence codes IEA (inferred from electronic annotation), NAS (non-traceable author statement) or RCA (inferred from reviewed computational analysis). GO terms annotated to <10 genes were excluded, leaving 8557 terms used in pathway analyses. Comparisons between gene or protein sets were made using the mouse Ensembl ID (63). Enrichment of gene sets derived from differential expression analysis for GO annotations, or other functionally defined gene sets, was determined by Fisher’s exact test, whereby all remaining tissue-expressed genes or proteins were used as the statistical background. Multiple testing was corrected for using the Bonferroni method.

Protein–protein interaction networks were compiled using GeneMANIA (64). Networks were filtered to include only physical interactions, and exclude interactions defined by co-expression, co-localization, shared domains or predictions.

### Mapping of Setd1a targets to genes

Data containing predicted Setd1a genomic binding sites were obtained from a recent study (14) of Setd1a targets in 6-week-old mouse prefrontal cortex using ChIP-seq. Setd1a peaks located at promoter or enhancer regions were mapped to genes using the mm10 mouse genome assembly. Peaks mapping to zero or multiple genes were excluded.

### Genetic association analysis

Recent schizophrenia case–control GWAS summary statistics were provided by the Psychiatric Genomics Consortium. The primary GWAS consisted of 69 369 cases and 94 015 controls of European or Asian descent (5). Single nucleotide polymorphisms (SNPs) with minor allele frequency >1% were annotated to genes using a 35 kb upstream/10 kb downstream window to allow for proximal regulatory regions. SNP association *P*-values were combined in MAGMA v1.08 (65) using the SNP-wise Mean model, controlling for linkage disequilibrium with the 1000 Genomes European reference panel (66). Gene set association analysis was performed using one-tailed competitive tests in MAGMA, conditioning on a background of tissue-expressed genes.

*De novo* coding variants observed in people diagnosed with schizophrenia were taken from published exome sequencing studies. In total, *de novo* variant data were derived from 3444 published schizophrenia-proband parent trios (67–76), as described previously (68,77,78). Gene set enrichment statistics were generated by a two-sample Poisson rate ratio test comparing the ratio of observed versus expected *de novo* variants in the gene set to a background set of genes. Expected numbers of variants were determined from per-gene mutation rates (79). The background set contained all tissue-expressed genes.

## Data availability

Transcriptomic data from RNA sequencing is available from the Gene Expression Omnibus with identifier GSE199428. The mass spectrometry proteomics data have been deposited to the ProteomeXchange Consortium via the PRIDE (80) partner repository with the dataset identifier PXD032742.

## Supplementary Material

Supplementary_Information_ddac105

Supplementary_Tables_ddac105
