# Supplementary material for: Developmental disruption to the cortical transcriptome and synaptosome in a model of SETD1A loss-of-function
Source: Hum Mol Genet. 2022 May 9;31(18):3095–106. doi: 10.1093/hmg/ddac105 (PMC9476630; doi:10.1093/hmg/ddac105)
Supplement: Supplementary_Information_ddac105 [file supplementary_information_ddac105.docx]

## Developmental disruption to the cortical transcriptome and synaptosome in a model of *SETD1A* loss-of-function

## Supplementary Information


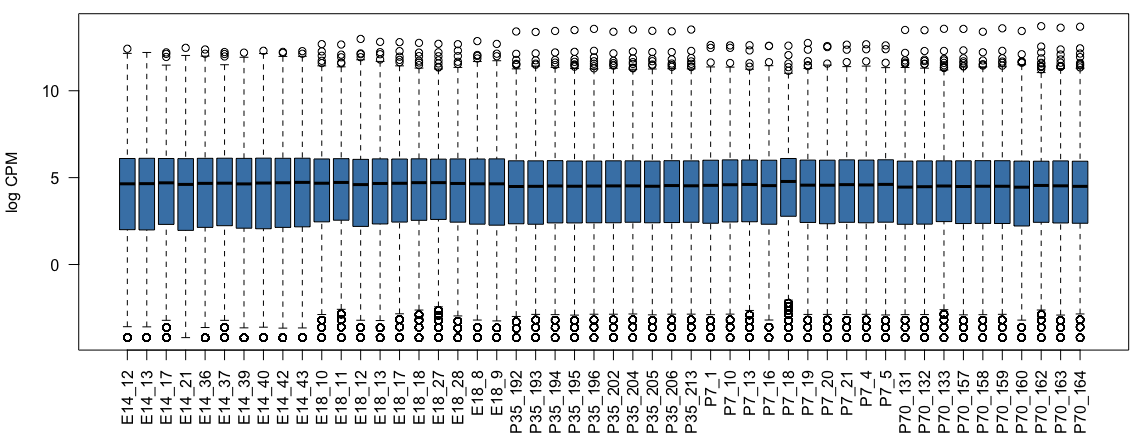


**Supplementary Figure S1** Read counts distribution following RNA sequencing of 50 cortical samples from wildtype mice and mice carrying a loss-of-function mutation in *Setd1a*, across five developmental stages. Data is presented in log_2_(counts per million).


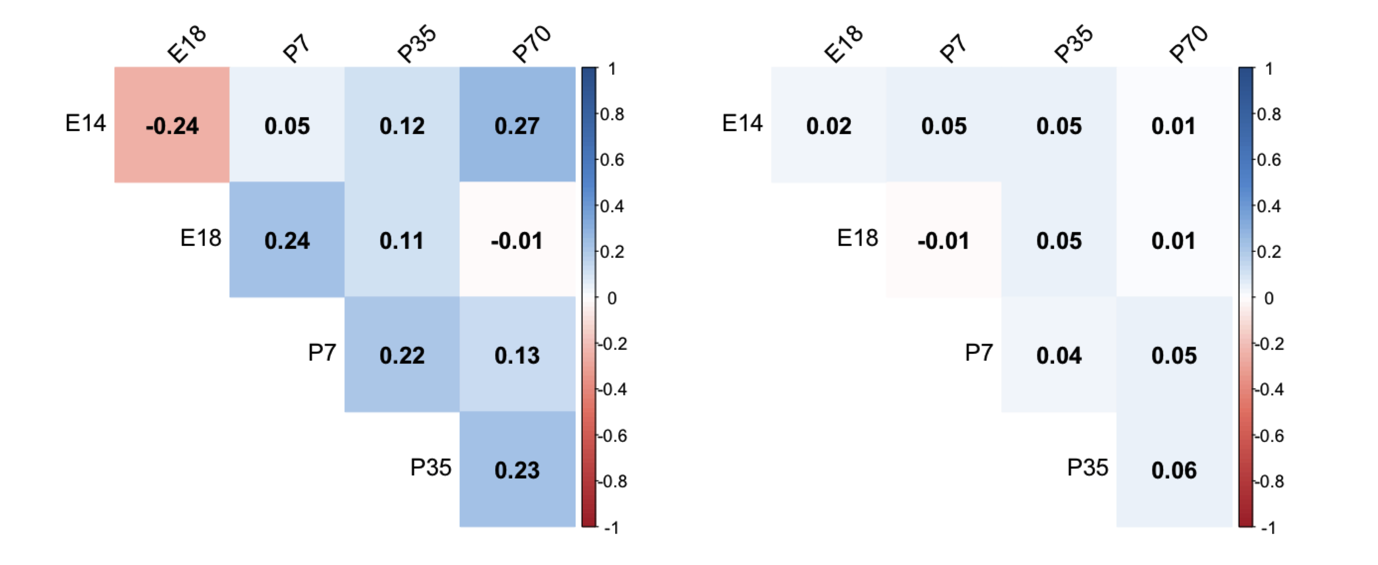


**Supplementary Figure S2** Comparisons of differential gene (left) or protein (right) expression across five developmental stages. Shown are pairwise Pearson correlation coefficients between the log fold change observed for each gene or protein in genotype contrasts at each stage. Colours indicate the strength and direction of the correlation.


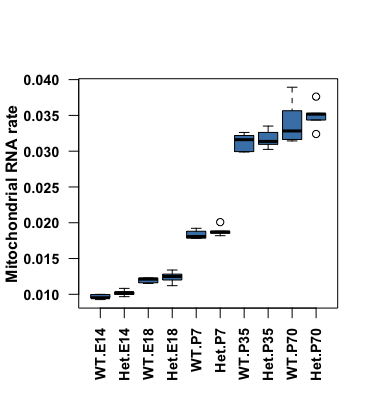


**Supplementary Figure S3** Comparison of the proportion of read counts aligned to the mitochondrial genome in cortical samples from wildtype and *Setd1a*^+/-^ mice at five stages of development. Wildtype (WT); Heterozygous (Het).


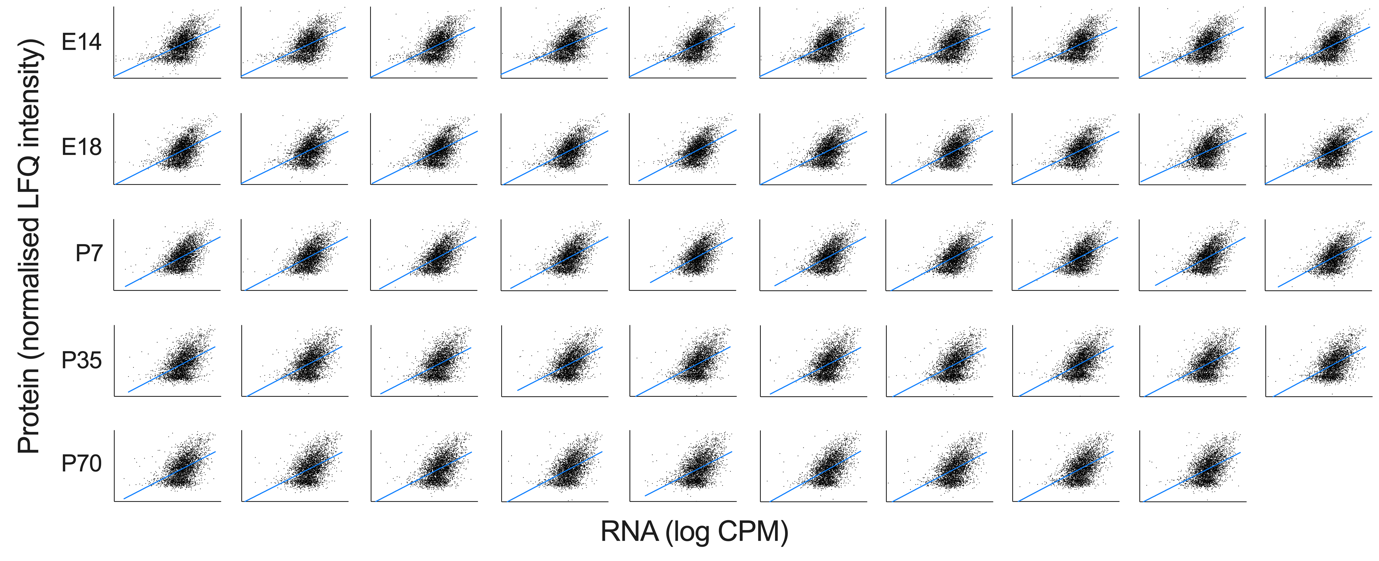


**Supplementary Figure S4** RNA-protein correlation of the synaptosome in 49 cortical samples. RNA sequencing of bulk tissue and mass spectrometry-based label-free quantitation of isolated synaptosomes was performed using tissue from frontal cortex of wildtype and *Setd1a*^+/-^ mice. One P70 sample processed for protein quantification was not subjected to RNAseq due to replacement during quality control. Median R^2^ = 0.24 ± 0.017.


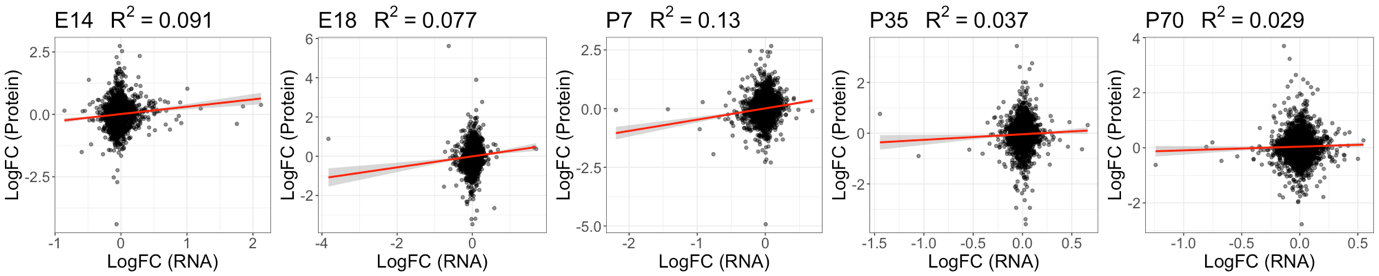


**Supplementary Figure S5** Comparisons of differential gene expression with differential protein expression at five developmental timepoints. Plotted are log fold changes for each synaptosomal gene and its corresponding protein following contrasts between cortical tissue from wildtype and *Setd1a*^+/-^ mice at each age. R^2^ values correspond to the Pearson correlation coefficient.

**Supplementary Figure S6** Schizophrenia genetic association of binned sets of proteins ranked by probability of differential expression in the synaptosome of frontal cortical tissue from *Setd1a*^+/-^ mice compared to wildtype controls. Each bin contains 50 genes. Shown is -log_10_(*P*-value) in one-tailed gene set enrichment analyses using common variants (left) or *de novo* rare variants (right).

**Supplementary Table S1** RNA sequencing sample metadata

| **Sample ID** | **Age** | **Genotype** | **RNA Integrity** | **Raw reads** |
| --- | --- | --- | --- | --- |
| E14_12 | E14 | WT | 8.1 | 79694358 |
| E14_13 | E14 | Het | 9.8 | 79911750 |
| E14_17 | E14 | Het | 9.9 | 84955826 |
| E14_21 | E14 | WT | 9.6 | 88454156 |
| E14_36 | E14 | Het | 9.5 | 86956670 |
| E14_37 | E14 | Het | 9.8 | 86827146 |
| E14_39 | E14 | WT | 10 | 93642210 |
| E14_40 | E14 | WT | 10 | 83172116 |
| E14_42 | E14 | Het | 9.9 | 94630422 |
| E14_43 | E14 | WT | 9.9 | 90555812 |
| E18_10 | E18 | Het | 9.8 | 83098980 |
| E18_11 | E18 | WT | 9.9 | 82988784 |
| E18_12 | E18 | Het | 8.1 | 84519088 |
| E18_13 | E18 | WT | 8.7 | 84856574 |
| E18_17 | E18 | Het | 6.8 | 79691188 |
| E18_18 | E18 | WT | 10 | 82975144 |
| E18_27 | E18 | WT | 9.7 | 84936680 |
| E18_28 | E18 | Het | 9.6 | 91058580 |
| E18_8 | E18 | Het | 7.8 | 85170458 |
| E18_9 | E18 | WT | 9.9 | 88822842 |
| P35_192 | P35 | Het | 8.8 | 96081558 |
| P35_193 | P35 | WT | 8.9 | 83699420 |
| P35_194 | P35 | Het | 8.9 | 86449832 |
| P35_195 | P35 | WT | 8.5 | 88274004 |
| P35_196 | P35 | Het | 8.6 | 82942578 |
| P35_202 | P35 | WT | 9 | 83955372 |
| P35_204 | P35 | Het | 8.7 | 81787242 |
| P35_205 | P35 | Het | 9.1 | 87622142 |
| P35_206 | P35 | WT | 8.9 | 83568088 |
| P35_213 | P35 | WT | 8.9 | 83351912 |
| P7_1 | P7 | Het | 9.7 | 82990994 |
| P7_10 | P7 | Het | 9.4 | 81501550 |
| P7_13 | P7 | WT | 9.6 | 85822912 |
| P7_16 | P7 | Het | 9.8 | 83329092 |
| P7_18 | P7 | WT | 9.7 | 85054368 |
| P7_19 | P7 | Het | 9.7 | 82828370 |
| P7_20 | P7 | WT | 9.6 | 82452790 |
| P7_21 | P7 | WT | 9.6 | 84390560 |
| P7_4 | P7 | WT | 9.9 | 82446960 |
| P7_5 | P7 | Het | 9.8 | 87511448 |
| P70_131 | P70 | Het | 9 | 83814238 |
| P70_132 | P70 | WT | 9 | 84029636 |
| P70_133 | P70 | Het | 8.4 | 83694650 |
| P70_157 | P70 | Het | 9.3 | 85776580 |
| P70_158 | P70 | WT | 6.7 | 86171434 |
| P70_159 | P70 | Het | 8.4 | 83081626 |
| P70_160 | P70 | WT | 8.5 | 84000502 |
| P70_162 | P70 | WT | 8.5 | 83499260 |
| P70_163 | P70 | WT | 8.7 | 89064386 |
| P70_164 | P70 | Het | 8.7 | 82100618 |
